# Cinnamon pretreatment modulates gene expression of tight junction proteins in a rat model of stroke

**DOI:** 10.22038/AJP.2024.24290

**Published:** 2024

**Authors:** Solmaz Najjary, Hossein Mostafavi, Hadi Feizi, Fatemeh Moradi, Mehdi Eskandari

**Affiliations:** *Department of Physiology, School of Medicine, Zanjan University of Medical Sciences, Zanjan, Iran*

**Keywords:** Cinnamon, Brain ischemia, Calpain I, Calpain II, Occludin, VEGF

## Abstract

**Objective:**

Brain ischemia generally results in irreversible brain damage or death. One of the most important features of an ischemic stroke is disruption of the Blood-brain barrier (BBB). In this study, we examined the effect of cinnamon hydroalcoholic extract consumption on BBB permeability and expression of some genes regulating its function.

**Materials and Methods:**

Sixty male Wistar rats were divided into 5 groups; sham (high-fat diet+ sham surgery), Model (Middle Cerebral Artery Occlusion, MCAO+ high-fat diet), Lovastatin (high-fat diet + lovastatin + MCAO surgery), low and high dosage cinnamon (high-fat diet + cinnamon 130 or 260 mg, respectively+ MCAO surgery). The two doses of cinnamon (130 and 260 mg) were administered intraperitoneally. Twelve hours after ischemic stroke induction, brain right hemisphere tissues were collected and *calpain I, calpainII, occludin* and *VEGF* genes expression were quantified by Real-Time -PCR. Accordingly, p-selection protein levels were measured by ELISA method.

**Results:**

Cinnamon hydroalcoholic extract reduced the BBB permeability compared with the model group (p<0.05). Stroke increased *calpain* and *VEGF* genes while decreased *occludin* gene expression (p<0.001). Conversely, cinnamon administration increased *occludin* gene expression while *calpain* and *VEGF* genes were down-regulated (p<0.01). Pretreatment with cinnamon significantly diminished the P-Selectin protein levels as compared with the model group dose dependently (p<0.001).

**Conclusion:**

It seems that cinnamon restores BBB function by regulating the elements involved in its permeability.

## Introduction

Stroke is the third leading cause of death and the most common cause of morbidity. Thus, preventive strategies against stroke and remediating subsequent disabilities are very important. Due to ischemic stroke, the blood-brain barrier (BBB) is disrupted, inflammation proceeds and this leads to damage of a significant number of neurons. Therefore, protecting the undergoing cells is so vital in the treatment of stroke (Pawluk et al., 2020). Following ischemic stroke, the integrity of the BBB is impaired leading to edema. Occludin is one of the proteins in the tight junctions of the BBB that plays an important role in maintaining its integrity at physiological and pathophysiological states (Pan et al., 2017; Yuan et al., 2020; Goncalves et al., 2022). On the other hand, calpain is a calcium-dependent cysteine protease that is present in almost all vertebrate cells and comes in two forms, calpain (calpain I) and mCalpain (calpain II), each responding to different concentrations of calcium (Zhang et al., 2017). Increased calpain activity destroys cytoskeletal proteins and the BBB and increases its permeability (Chun and Prince, 2009). Vascular endothelial growth factor (VEGF) is an effective mediator of vascular permeability and neuroprotection (Babkina et al., 2022). It is notable that ischemic condition and low tissue oxygen pressure prompts VEGF expression in endothelial cells leading to plasma extravasation and thus edema (Chen et al., 2021). In recent years, the use of neuroprotective drugs in ischemic stroke is a point of focus. Despite many studies which have been done about neuroprotective herbs, their efficacy in most cases has not been proven yet. Cinnamon is one of these herbs found mainly in tropical regions such as southern India and Sri Lanka. Cinnamon is a very well-known and widely used herb in traditional medicine. *Cinnamomum zeylanicum* and *Cinnamomum cassia* are among the main species (Yanakiev, 2020). The major constituents of cinnamon includes cinnamaldehyde, eugenol, safrole and volatile oils (Rao and Gan, 2014). Cinnamon and its active compounds have anti-diabetic, antioxidant, anti-inflammatory and anti-angiogenic properties and can prevent damage to the nervous system following diseases such as Parkinson's, Alzheimer's and stroke (Momtaz et al., 2018). In addition, new findings have shown that cinnamon and its active compounds can significantly reduce oxidative damage in ischemic brain. Cinnamon, inhibits the expression of cyclooxygenase-2 (*COX-2*) -encoding genes by its anti-inflammatory properties and prevents the conversion of phospholipase A2 to thromboxane A2 (TxA2). It also reduces the formation of blood clots created in blood vessels (Rao and Gan, 2014). 

Previously, the neuroprotective properties of cinnamon in animal models of stroke and consequent BBB degeneration have been demonstrated (Mostafavi, 2023). However, the real mechanisms involved in these effects have not been clarified yet. To date, some proteins which are crucial to BBB integrity have been identified (Pan et al., 2017; Zhang et al., 2017). Nevertheless, the effects of cinnamon on gene expression of these proteins have not been studied. Therefore, the aim of the present study was to determine the effect of pretreatment of cinnamon hydro-alcoholic extract on the expression of *calpain*, *occludin* and *VEGF* genes and P-Selectin protein levels in injured brain tissue.

## Materials and Methods

### Preparation of a high-fat diet (HFD) and extract of cinnamon

The HFD was prepared according to our previous research (Mostajabi et al., 2022). The hydroalcoholic cinnamon extract and lovastatin powder were obtained from Golestan Company (Tehran, Iran) and Osveh Pharmaceutical Company (Tehran, Iran), respectively. 

For this study, 60 adults male Wistar rats aged approximately 4 to 6 weeks with an initial weight of 160-180 g were obtained from Pasteur Institute (Tehran, Iran). The rats were kept in suitable living conditions with 12:12 hr day night cycle at room temperature (22-24°C), appropriate humidity and free access to water and food. The animals were randomly selected and divided into 5 groups; Sham group (receiving HFD for 8 weeks and surgery stress), middle cerebral artery occlusion (MCAO) model group (Rats receiving HFD for 8 weeks and undergoing MCAO surgery), Lovastatin group (Rats receiving HFD for 8 weeks and lovastatin injection (10 mg/kg) (Jadhav et al., 2006) for 6 weeks, undergoing MCAO surgery, positive control), Low-dose cinnamon group (Rats receiving HFD for 8 weeks and 130 mg of cinnamon hydroalcoholic extract injection for 6 weeks, then subjected to MCAO surgery) (Ranasinghe et al., 2013), High-dose cinnamon group (Rats receiving HFD for 8 weeks and injected with 260 mg of hydroalcoholic cinnamon extract for 6 weeks and then subjected to MCAO surgery). Ethical approval was obtained from the Ethics Committee on Animal Experimentations of Zanjan University of Medical Sciences, Zanjan, Iran (Ethical No. ZUMS.REC.1396.153).

### Induction of the transient ischemia-reperfusion (I/R) rat model

The transient I/R model induction was performed as described by Longa et al. (Longa et al., 1989). The animals were anesthetized with an intraperitoneal injection of with 10% chloral hydrate at a dose of 350 mg/kg (Merck, Germany, Cat No. 102425). After anesthetic induction, the tip of a silicon-coated commercial 3-0 monofilament was advanced long past the carotid bifurcation into the internal carotid artery lumen through the external carotid artery stump, and to occlude the origin of the middle cerebral artery (MCA) until mild resistance was felt. The filament was kept in place for 1 hr and then withdrawn to perform the reperfusion operation. Afterwards, the rats were allowed to regain consciousness. In the sham group, the rats were subjected to the operation identically, apart from filament insertion and middle cerebral arteries occlusion.

### Measurement of BBB permeability

Evans Blue (EB) leakage was used to evaluate BBB permeability. After 60 min of ischemia, 4 ml/kg of 2% EB solution was injected through the animals’ tail vein. To remove EB from the vessel, 250 ml saline was infused through the left ventricle. Then, the brain tissue was homogenized in 2.5 ml phosphate buffered saline, and then 2.5 ml of trichloroacetic acid 60% (Merck, Germany) was added to precipitate the protein content. The supernatants were measured at 610 nm for the absorbance of EB using a spectrophotometer (UV-Visible, USA). The results are expressed as micrograms per gram of the brain tissue calculated according to a standard curve (Mostajabi et al., 2022).

### Real-time quantitative polymerase chain reaction (RT-qPCR) for measuring gene expression

To extract total RNA, approximately 100 mg of right brain hemisphere tissue was homogenized in 1 ml of triazole solution (Sinacolon, Iran). After five minutes, 200 µl of chloroform was added to the samples and vortexed for 15 sec. The samples were incubated on ice for 10 min and then centrifuged at 12000 g and 4°C for 15 min. The upper colorless phase (containing RNA) was separated and 500 μl of isopropanol was added to it. The samples were again incubated on ice for 10 min and placed in a refrigerated centrifuge for 12 min at 12,000 g and 4°C. The precipitate containing RNA was mixed with 1 ml of 75% ethanol and centrifuged for 5 min at 7500 g and 4°C. Next, the topmost fluid phase was discarded, 20 µl of TE buffer was added to the samples and the samples were incubated at 60°C in Ben Marie for 10 min to dissolve the extracted RNA. Spectrophotometry was used to measure RNA concentration. A260/A280 ratio of 1.8-2.1 was considered indicative of purified RNA. The cDNA (complementary DNA) was synthesized using 500 ng of RNA, Random Hexamer Primer and Reverse Transcriptase enzyme. The expression levels of *occludin*, *calpain* *I*, *calpain II* and *VEGF* genes were measured by quantitative real-time PCR using Syber green (Takara Primix). The final volume of the reaction mixture was 10 µl and each reaction was performed in triplicate. Primers were designed using Gene Runner and Oligo Analyzer software and *β-actin* gene was used as internal control. The sequence of primers used are depicted in Table 1. The cycling conditions were: initial denaturation at 95°C for 30 sec, followed by 40 cycles of 95°C for 5 sec, and 59.3, 61.5, 58.1, 60.4 and 62°C (respectively for *calpain I*, *calpain II*, *occluding*, *VEGF* and *β-actin* genes) for 34 sec, and 72°C for 30 sec in sequence.

### ELISA test for measuring P-Selectin protein levels

Tissue samples were weighed after being defrozen. Next, they were homogenized with 1 ml of phosphate buffered saline (PBS) buffer and then centrifuged for 20 min at 3000 g and 4°C. The obtained supernatant was kept at -20°C until analyzed by ELISA test. P-Selectin protein levels were measured using ELISA kit (Rat P-Selectin Elisa Kit/ Cat. No. E0074Ra. Shanghai, China, Biotech Co) according to the instructions provided. Protein content is expressed as ng/mg tissue. 

### Data analysis

In order to investigate the effect of cinnamon extract on the relative gene expression levels, first the normality of the data was analyzed by Smirnov Kolmogorov test and then the results were analyzed by one-way ANOVA and Tukey’s *post hoc* test. A p<0.05 was considered statistically significant.

## Results

**Effects of the cinnamon extract on BBB**

**Figure 1:**
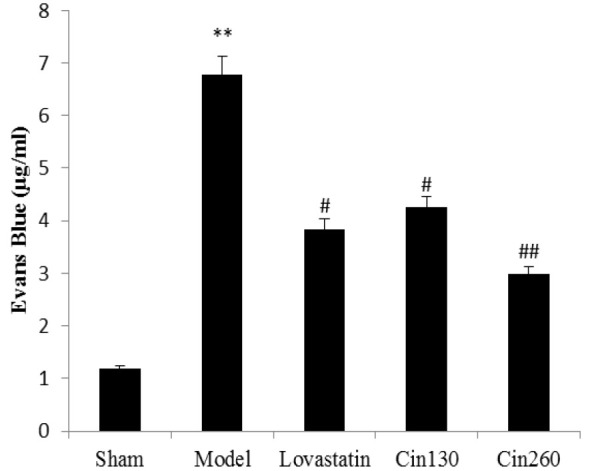
Comparison of the Average Permeability of the BBB in the Damaged Hemisphere in Sham, Model, Lovastatin, Low dose Cinnamon (Cin 130) and High-dose Cinnamon (Cin 260) Groups. Cin: Cinnamon; BBB: Blood-brain barrier; SEM: Standard error of the mean. The data are expressed as the mean±SEM. #(p<0.05) and ##(p<0.01) compared with the model group, **(p<0.01) compared with the sham group (n=6).

As presented in Figure 1, the highest BBB permeability to EB was seen in the ischemic hemisphere of the model group animals compared to the sham group (p<0.01). Cinnamon and lovastatin treatment significantly decreased the permeability to the dye as comparing with the model group (p<0.05). However, high-dose cinnamon (Cin 260) diminished it more significantly (p<0.01).

**Quantitative measurement of** ***calpain I*** **,** ***calpain II*** **,** ***occludin*** **and** ***VEGF*** **gene expression**

**Figure 2:**
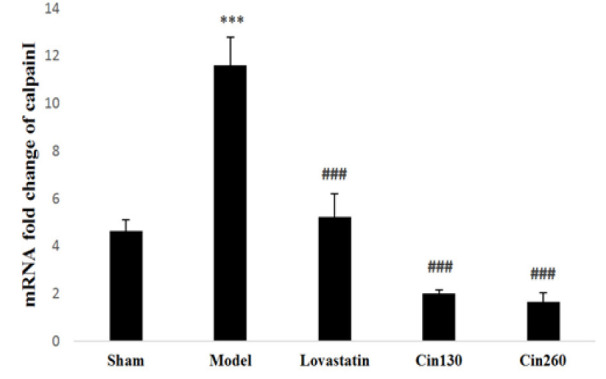
*Calpain I* gene expression in ischemic hemispheres at the end of study in the rats of groups Sham, Model, Lovastatin, and treated with cinnamon 130 (Cin130) and cinnamon 260 (Cin260). ***(p<0.001) compared with the sham group. ###(p<0.001) compared with the model group. All the results are reported as Mean±SEM. (n=6).

As shown in Figure 2, *calpain I* gene expression in the ischemic hemispheres of the model group was increased compared with the sham operated animals (p<0.001). Treatment by lovastatin and both cinnamon doses significantly decreased the *calpain I* gene expression (p<0.001). Expression levels of this gene were not significantly different between the treatment groups receiving low-dose and high-dose of cinnamon and the lovastatin group.

**Figure 3:**
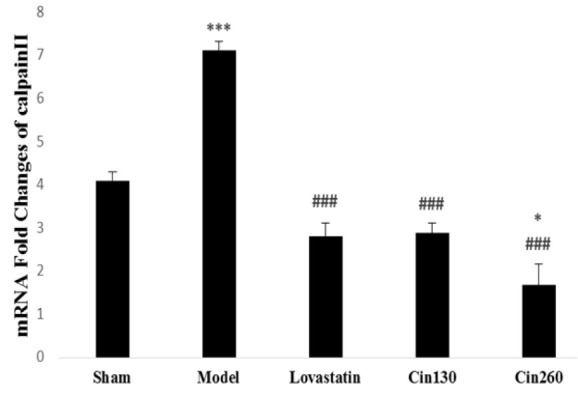
*Calpain II* gene expression in ischemic hemispheres at the end of study in the rats of groups Sham, Model, Lovastatin, and treated with cinnamon 130(Cin130) and cinnamon 260(Cin260). ***(p<0.001) compared with the sham group. ###(p<0.001) compared with the model group. *(p<0.05) compared with the cin130 and Lovastatin groups. All the results are reported as Mean±SEM. (n=6).

The expression level of *calpain II* mRNA was significantly increased after stroke induction (p<0.001). Six weeks of pretreatment with hydroalcoholic extract of cinnamon significantly decreased the *calpain II* mRNA expression compared with the model group (p<0.001). Expression levels of this gene in the high-dose cinnamon group was significantly lower than that of the lovastatin and low-dose cinnamon (p<0.05) (Figure 3).

**Figure 4:**
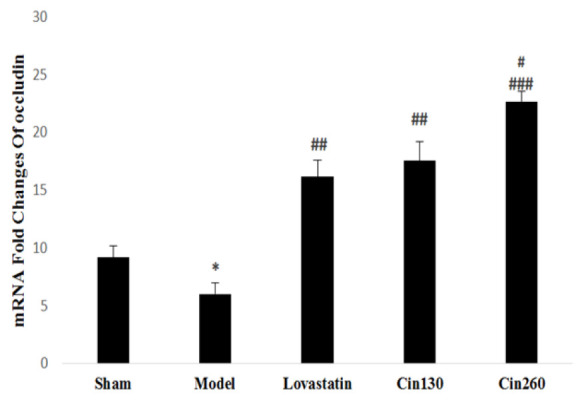
*Occludin* gene expression in ischemic hemispheres at the end of study in the rats of groups sham, Model, Lovastatin, and treated with cinnamon 130 (Cin130) and cinnamon 260 (Cin260). * (p<0.05) compared with the Sham group. ##(p<0.01) and ###(p<0.001) compared with the model group. #(p<0/05) compared with the cin130 and lovastatin groups. All the results are reported as Mean±SEM. (n=6).

As shown in Figure 4, *occludin* gene expression in the ischemic hemispheres of the model group was decreased compared with the sham group. Pretreatment with lovastatin and hydroalcoholic extract of cinnamon, elevated the *occludin* gene expression as compared with the model group (p<0.01) and (p<0.001). Expression levels of this gene in the high-dose cinnamon group was significantly higher than that of the cin130 and lovastatin groups (p<0.05).

**Figure 5:**
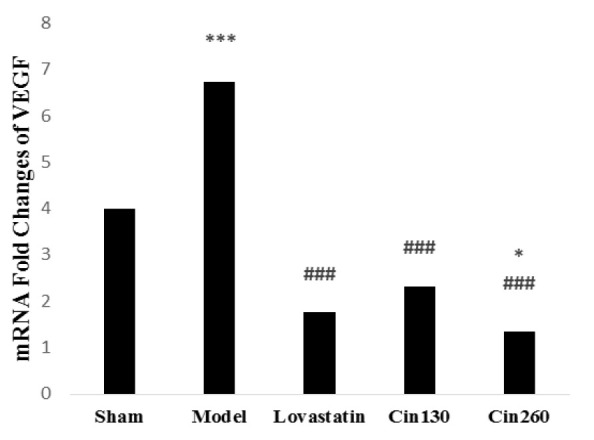
*VEGF* gene expression in ischemic hemispheres at the end of study in the rats of groups Control, Sham, Model, Lovastatin, Vehicle and treated with cinnamon 130 (Cin130) and cinnamon 260 (Cin260). ***(p<0.001) compared with the sham group. ###(p<0.001) compared with the model group. *(p<0.05) compared with the cin130 group. All the results are reported as Mean±SEM. (n=6).

*VEGF* gene expression level was significantly increased following the induction of ischemic stroke (1hI /12hR) (p<0.001). Six weeks of pretreatment with lovastatin and both cinnamon doses significantly reduced the *VEGF* mRNA expression compared with the model group (p<0.001). This reduction was dose dependent in cinnamon-treated groups (p<0.05) (Figure 5).

**P-Selectin protein level**

**Figure 6:**
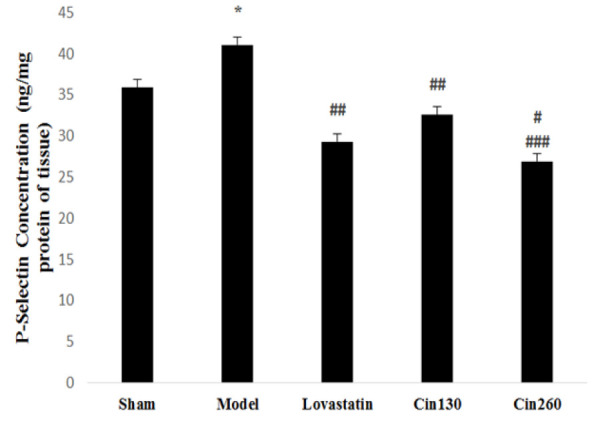
P-selectin protein assessment by ELISA method in ischemic hemispheres at the end of study in the rats of groups Sham, Model, Lovastatin, and treated with cinnamon 130 (Cin130) and cinnamon 260(Cin260). *(p<0.05) compared with the sham group. ##(p<0.001) and ###(p<0.001) compared with the model group. #(p<0.05) compared with the cin130 group. All the results are reported as Mean±SEM. (n=6).

The P-Selectin protein level in the model group increased significantly compared to the sham group (p<0.05). Pretreatment with lovastatin and cinnamon hydroalcoholic extract significantly diminished the P-Selectin protein levels as compared with the model group (p<0.01 and p<0.001, respectively). The amount of P-Selectin protein in brain tissue was lower in the high-dose cinnamon group than that of the low-dose cinnamon group (p<0.05). P-Selectin levels were not significantly different in the treatment groups receiving low-dose and high-dose of cinnamon extract compared to the lovastatin group (Figure 6).

## Discussion

Previously, we have shown that pretreatment with cinnamon prevents stroke-induced BBB integrity loss and restores its permeability (Mostafavi et al., 2023). This finding was similar to that found by Yulug et. al (Yulug et al., 2018). To find out the mechanisms in details, in the present study, we examined the expression of some genes related to BBB integrity. As indicated in our results, expression levels of *calpain I* and *calpain II* genes were significantly increased in the MCAO model group. In physiological conditions, calpastatin endogenously regulates calpain activity (Moghadam et al., 2018; Sternberg and Schaller, 2020). However, throughout brain ischemia, calcium level rises and glutamate receptors are triggered. This counteracts endogenous inhibitory systems and causes calpain over activation. Studies by Kinga et al. and Mahmood Q et al. in 2010 and 2017 respectively, showed that due to stroke, calcium enters intracellular space and stimulates calpain activity (Mahmood et al., 2017; Jarrahi et al., 2020). A couple of studies have revealed that calpain regulates matrix-metalloproteinase activity positively (Tsubokawa et al., 2006; Jiang et al., 2012). It has been shown that matrix metalloproteinase-9 (MMP-9) was activated 4 hr to 4 days after induction of MCAO and that plasma MMP-9 levels were significantly increased in acute ischemic stroke. These studies suggest that inhibition of MMP can have a beneficial effect in preventing stroke complications, but its effect depends on the timing of treatment and the severity of the brain injury (Lakhan et al., 2013). Some studies have shown that matrix metalloproteinase increases rapidly following acute ischemic stroke. This causes endothelial cell membrane and occludin degradation in BBB leading to edema (Zhang et al., 2017; Yuan et al., 2020). Liu et al. reported that inhibition of MMP-9 prevents the damage to the brain by maintaining BBB integrity (Liu et al., 2009). *In vitro* studies have shown that occludin is a suitable substrate for matrix metalloproteinases, especially MMP-9. Thus, an increase in its activity leads to occludin degradation and increased BBB permeability (Liu et al., 2009; Rong et al., 2021). In a study in 2013, the synthetic inhibitor MMPs (1101-BB) prevented the disruption of BBB through preventing the destruction of occluding (Lakhan et al., 2013). Since, in our research, we have shown that cinnamon downregulates the *calpain* gene expression, it seems that cinnamon may affect the occludin reestablishment through both calpain itself or MMPs activity.

In a study in 2017, Xu et al. showed that ischemic injury decreases occludin expression in the cerebral cortex while melatonin protects the brain against ischemic injury by decreasing *AQP4* and increasing *occludin* genes expression in the brain tissue (Xu et al., 2017). In our findings also *occludin* gene expression was decreased following induction of ischemic stroke. Notably, cinnamon pretreatment increased *occludin* gene expression in injured animals. To date, we have not found any report about the effect of this herbal product on *occludin* gene expression in the brain but some studies have indicated this effect of cinnamon in the intestinal tight junctions (Sun et al., 2017; Kim and Kim, 2019). On the other hand, Pan et al. believe that plasma occludin increase could be supposed as a biomarker of BBB destruction in the acute phase of ischemic stroke. They reported that following ischemic stroke, occludin was broken into two segments of different sizes (31 and 55 kDa) and its amount maintains high until 24 hr (Pan et al., 2017). 

The results of this study showed that *VEGF* gene expression was significantly increased after induction of ischemic stroke compared to the sham group. VEGF is one of the most important specialized angiogenic regulators. Leading to specific types of MMPs secretion from the endothelial cells, it breaks down the basement membrane. Consequently, endothelial cells migrate and proliferate and angiogenesis is developed (Martínez, 2006). As a result of angiogenesis, BBB permeability increases and aggravates brain damage in the acute phase of the stroke. However, it protects neuronal function in the chronic phase of the stroke (Mullen et al., 2012, Rust, 2020). Jiang et al. showed that increased *VEGF* gene expression in the acute phase of stroke causes small vessel leakage which leads to cerebral edema and increased stroke-induced damage (Jiangt et al., 2014). Zhang et al. showed that VEGF expression increases around the stroke center three hours after ischemia causing vascular permeability and impaired BBB function (Zhang et al., 2017). Hypoxia is one of the most important triggers for VEGF expression. Hypoxic conditions stimulate hypoxia inducible factor 1 subunit alpha (HIF1-α) expression. This factor further induces the expression of the *VEGF* gene by activating the Akt/PI3K or the ERK/MEK pathways (Zachary and Gliki, 2001; Egginton, 2009). Mu et al. have found that 1.5 hour after induction of stroke, *HIF1-α* gene expression increased and lead to increased *VEGF* gene expression (Mu et al., 2003). Zhang et al. (2016) also have reported *HIF1-α* and *VEGF* gene expression increment following induction of MCAO stroke (Zhang et al., 2016). Several other studies have been carried out confirming these results, all indicating the key role of *HIF1-α* in *VEGF* gene expression (Ramakrishnan et al., 2014; Chen et al., 2020).

In the present study, pretreatment with hydroalcoholic extract of cinnamon significantly reduced the expression of *VEGF* gene in a dose-dependent manner. Based on this result, it looks that VEGF inhibition by cinnamon in acute phase of stroke decreases permeability of BBB and decreases the damage caused by stroke. Kwon et al. showed that aqueous extract of cinnamon inhibits tumor growth and angiogenesis by inhibiting HIF1-α and COX-2 (Kwon et al., 2009). Bae et al concluded that cinnamic aldehyde found in cinnamon inhibited HIF1-α and consequently inhibited VEGF expression in cancer cells (Bae et al., 2015). Zhang in 2017, in line with the results of previous studies, showed that aqueous extract of cinnamon inhibits HIF1-α -induced *VEGF* gene expression (Zhang et al., 2017). In the present study, the hydroalcoholic extract of cinnamon was probably involved in the reduction of *VEGF* gene expression through inhibition of HIF1- α.

Tight junctions in the BBB contain a protein complex that holds the plasma membrane of the endothelial cells tightly together, limiting the paracellular transport of substances between the blood and the brain. Neurodegenerative diseases, such as stroke, are followed by a disrupted BBB which allows the entry of inflammatory factors produced during reperfusion (Tabassum et al., 2015; Pawluk et al., 2020). As inflammatory factors, such as interleukin 6 (IL-6) and tumor necrosis factor α (TNFα), enter the blood vessels, the recruitment of leukocytes at the site of injury occurs by adhesion molecules including selectin. Selectins are a family of membrane glycoproteins that contain 3 molecules of P, E, and L selectin (McEver, 2015). The P-Selectin molecule is responsible for initiating adhesion of leukocytes to vascular endothelium and plays an important role in the interaction between leukocytes and endothelial cells in inflammatory processes (Furie and Furie, 2004; McEver, 2015). In the present study, P-Selectin protein levels in the model group were significantly increased compared to the control group. Nadar et al. reported in 2004 that plasma levels of P-Selectin protein increased after MCAO stroke. While its antagonist was able to decrease P-Selectin protein levels (Nadar et al., 2004). The results of this study were similar to those of Htun et al. in 2006, who reported that P-selectin protein levels increased in the acute phase of stroke (Htun et al., 2006). In this study, two doses of cinnamon were used as pretreatment. The results showed that both doses of cinnamon significantly reduced the P-Selectin protein levels compared to the model group. Zhao et al. in 2015 have shown that cinnamaldehyde in cinnamon suppresses inflammatory responses after ischemic stroke by reducing the release of inflammatory mediators and leukocyte infiltration (Zhao et al., 2015). Additionally, it was found that cinnamaldehyde inhibits inflammation by down regulating COX-2 and *TNF* gene expression and inhibiting NF-κB and the p53 pathway (Almoiliqy et al., 2020). According to the results of these studies, it seems that cinnamon has been effective in reducing the P-Selectin levels by inhibiting inflammatory mediators.

Overall, the findings of this study suggest that pretreatment of cinnamon extract can exert protective effects on the stroke-induced BBB disruption, decreasing its permeability and thus reducing the risk of ischemic edema and subsequent neuronal damage.

**Table 1:** The primers used in this study

Gene	Product size (bp)	Primer sequence	Accession number
*Calpain I*	101	F: 5'-TTTGGCCGGGACATGGAGA-3' R: 5'-TTGGCCAGGAAGAAGTCACG-3'	XM_039103034.1
*Calpain II*	103	F: 5'-ATCTCTGCCTTTGAGCTGCA-3' R: 5'-CCACCATGATCTTACAGGTCTCG-3'	NM_017116.2
*Occludin*	98	F: 5'-CTGTCTATGCTCGTCATCG-3' R: 5'-CTCCAAAGATGCCCGTTCCA-3'	XM_039103245.1
*VEGF*	102	F: 5'- CATGCGGATCAAACCTCACC-3' R: 5'-TCTGGCTTTGTTCTATCTTTCTTTGG-3'	NM_001287114.1
*β-actin*	250	F: 5'-CATGTACGTTGCTATCCAGGC-3' R: 5'-CTCCTTAATGTCACGCACGAT-3'	NM_031144.3
